# Pyogenic Sacroiliitis in a 13-Month-Old Child: A Case Report and Literature Review

**DOI:** 10.1097/MD.0000000000001581

**Published:** 2015-10-23

**Authors:** Leroux Julien, Bernardini Isabelle, Grynberg Lucie, Grandguillaume Claire, Michelin Paul, Ould Slimane Mourad, Nectoux Eric, Deroussen François, Gouron Richard, Angelliaume Audrey, Ilharreborde Brice, Renaux-Petel Mariette

**Affiliations:** From the Clinique Chirurgicale Infantile, CHU de Rouen, Hôpital Charles Nicolle, Rouen cedex (LJ, BI, GL, R-PM); Université de Rouen, Mont-Saint-Aignan (LJ, BI, GL, GC, R-PM, OSM); Département d’Anesthésie et Réanimation, CHU de Rouen, Hôpital Charles Nicolle (GC); Service de Radiologie, CHU de Rouen, Hôpital Charles Nicolle (MP); Service de Chirurgie Orthopédique et Traumatologique, CHU de Rouen, Hôpital Charles Nicolle, Rouen cedex (OSM); Service de Chirurgie et d’Orthopédie de l’Enfant, CHRU de Lille, Lille cedex (NE); Université Lille 2 Droit et Santé, Lille (NE); Service de Chirurgie de l’Enfant, CHU Amiens-Picardie site sud, Amiens cedex 1 (DF, GR); Université de Picardie, Amiens (DF, GR); Service de Chirurgie Infantile, CHU de Bordeaux, Hôpital des Enfants, place Amélie Raba-Léon Bordeaux (AA); Université de Bordeaux, Collège Sciences de la Santé, Bordeaux cedex (AA); Service d’Orthopédie Pédiatrique, Assistance Publique des Hôpitaux de Paris, Hôpital Robert Debré, Paris (IB); and Université Paris Diderot Paris 7, Paris, France (IB).

## Abstract

Pyogenic sacroiliitis is exceptional in very young children. Diagnosis is difficult because clinical examination is misleading. FABER test is rarely helpful in very young children. Inflammatory syndrome is frequent. Bone scintigraphy and MRI are very sensitive for the diagnosis. Joint fluid aspiration and blood cultures are useful to identify the pathogen. Appropriate antibiotic therapy provides rapid regression of symptoms and healing. We report the case of pyogenic sacroiliitis in a 13-month-old child.

Clinical, biological, and imaging data of this case were reviewed and reported retrospectively.

A 13-month-old girl consulted for decreased weight bearing without fever or trauma. Clinical examination was not helpful. There was an inflammatory syndrome. Bone scintigraphy found a sacroiliitis, confirmed on MRI. Aspiration of the sacroiliac joint was performed. Empiric intravenous biantibiotic therapy was started. Patient rapidly recovered full weight bearing. On the 5th day, clinical examination and biological analysis returned to normal. Intravenous antibiotic therapy was switched for oral. One month later, clinical examination and biological analysis were normal and antibiotic therapy was stopped.

Hematogenous osteoarticular infections are common in children but pyogenic sacroiliitis is rare and mainly affects older children. Diagnosis can be difficult because clinical examination is poor. Moreover, limping and decreased weight bearing are very common reasons for consultation. This may delay the diagnosis or refer misdiagnosis. Bone scintigraphy is useful to locate a bone or joint disease responsible for limping. In this observation, bone scintigraphy located the infection at the sacroiliac joint. Given the young age, MRI was performed to confirm the diagnosis. Despite the very young age of the patient, symptoms rapidly disappeared with appropriate antibiotic therapy.

We report the case of pyogenic sacroiliitis in a 13-month-old child. It reminds the risk of misdiagnosing pyogenic sacroiliitis in children because it is exceptional and clinical examination is rarely helpful. It also highlights the usefulness of bone scintigraphy and MRI in osteoarticular infections in children.

## INTRODUCTION

Pyogenic sacroiliitis is an osteoarticular infection particularly rare in children and exceptional in very young children. It reaches then preferably older children.^1–5^

Clinical presentation may be poor and misleading, especially in young children, so that diagnosis can be difficult.^6^ Most often, children consult for pain or functional impairment. Fever is inconstant. The most sensitive clinical sign is the FABER test: pain is caused or exacerbated at the sacroiliac joint when positioning the contralateral hip in flexion (F), abduction (AB), and external rotation (ER).^3,7^

There is most often an inflammatory syndrome with moderate increased protein C reactive (PCR).

X-rays are usually normal.^6^

Bone scintigraphy and magnetic resonance imaging (MRI) are particularly sensitive for the diagnosis of sacroiliitis but MRI has the advantage of being helpful to search an abscess that should be drained.^4,5,8–14^

Joint fluid aspiration and blood cultures are useful to identify the pathogen.^1,7^ Staphylococcus aureus is the main pathogen responsible for pyogenic sacroiliitis.^1^

Appropriate antibiotic therapy provides rapid regression of symptoms and healing.^8^ This observation reports a rare case of pyogenic sacroiliitis in a very young child. It focuses on difficulties to make the diagnosis in young children, reminds the risk of misdiagnosis, and highlights the usefulness of bone scintigraphy and MRI in osteoarticular infection in children.

No ethical approval was necessary since we report a retrospective case report but informed consent was given by parents.

## CASE REPORT

A 13-month-old girl consulted the pediatric emergency for decreased weight bearing on her right lower limb worsening for 4 days without fever or trauma. She had no noticeable antecedent. Temperature was 37.6°C. There was neither reduction of range of motion of hips or knees, nor pain on palpation of femoral or tibial metaphysic, nor spinal stiffness. The FABER test did not provide information given the very young age of the patient. Antero-posterior (AP) X-ray of the pelvis was normal. Biology found an inflammatory syndrome with a PCR = 38 mg/L and leukocytes = 18.8 G/L with 47% of polynuclear neutrophils.

**FIGURE 1:**
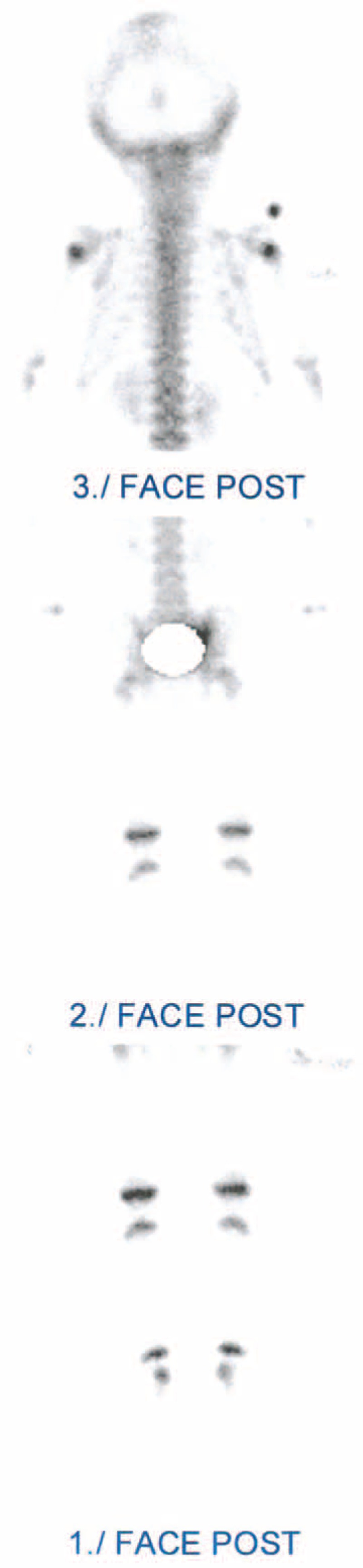
Posterior view of bone scintigraphy. Hypermetabolism of the right sacroiliac joint.

**FIGURE 2:**
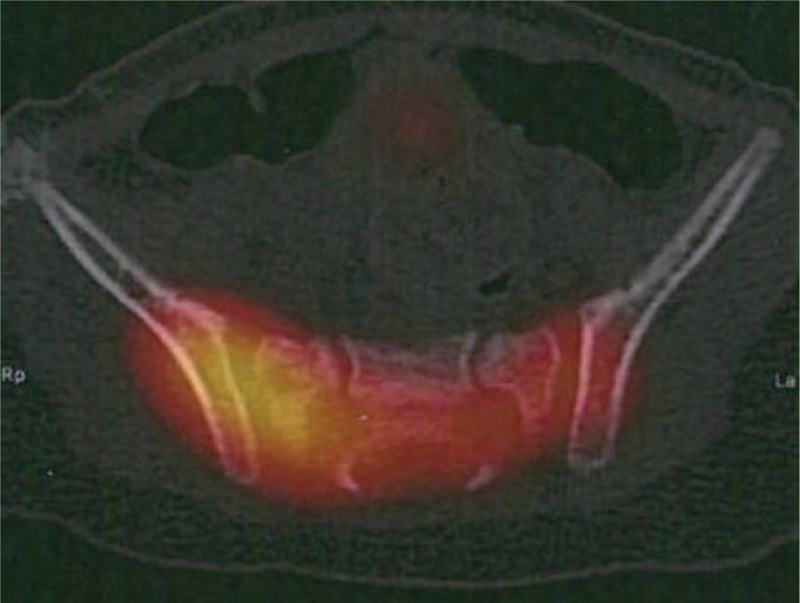
Axial view of SPECT. Hypermetabolism of the right sacroiliac joint.

**FIGURE 3:**
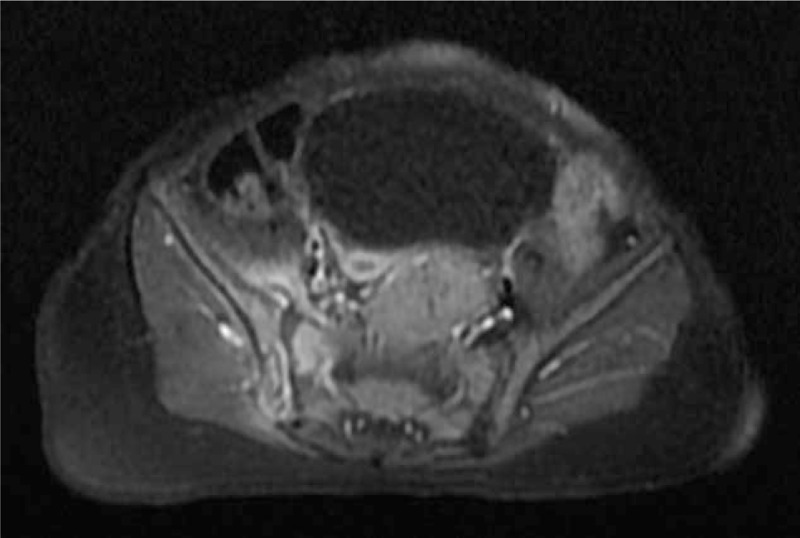
Axial T1 weighted with gadolinium of the pelvis. Hyperintense signal of ilium and sacrum on each side of the right sacroiliac joint and effusion (more noticeable at the anterior aspect of the sacroiliac joint) are typical of sacroiliitis.

Because of the functional impairment, the inflammatory syndrome, and the absence of painful site, a bone scintigraphy (Fig. 1) completed with a single-photon emission computed tomography (SPECT) (Fig. 2) was performed and found a right sacroiliitis. Given the young age of the patient, an MRI was performed to confirm the diagnosis. Inflammation of ilium and sacrum on each side of the right sacroiliac joint and effusion were suggestive of sacroiliitis (Fig. 3). An aspiration of the right sacroiliac joint was performed to try to identify the pathogen. An empiric intravenous biantibiotic therapy with cefotaxim and gentamycin was started. The next day, the patient recovered full weight bearing on the right lower limb. Bacteriological analysis found a Staphylococcus aureus susceptible to methicillin.

On the 5th day, clinical examination was normal and there was no inflammatory syndrome with a PCR <5 mg/L. Intravenous antibiotic therapy was switched for oral antibiotic therapy with amoxicillin and clavulanic acid for 4 weeks. At 1 month of starting the treatment, clinical examination and biological analysis were normal and antibiotic therapy was stopped.

## DISCUSSION AND REVIEW OF LITERATURE

In 1980, Schaad et al made a large review of literature about pyogenic sacroiliitis in 77 children. But nowadays, pathogens, imagery, and treatment have changed so that we realized a new review of cases and series published since 1980 to discuss this report. We find 11 articles that represent 78 patients (Table 1 ).^2–4,7,14–20^

**Table 1:**
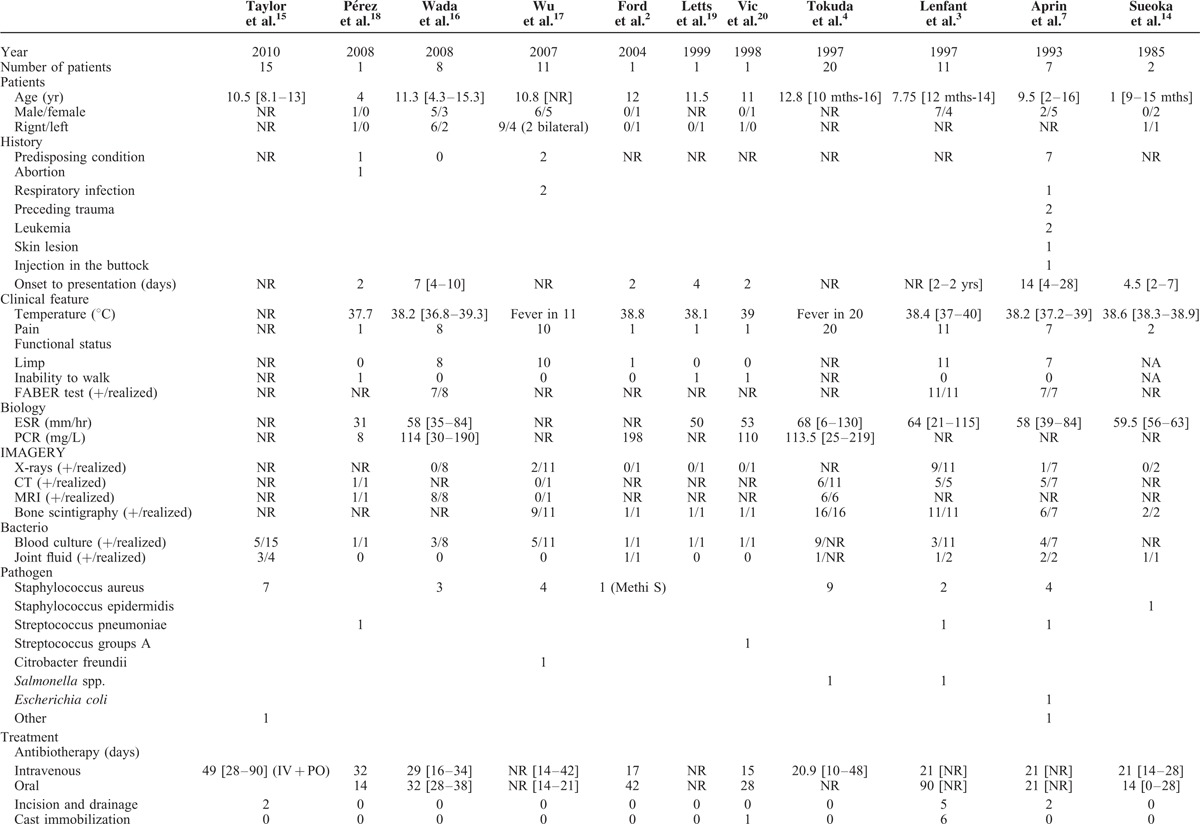
History, Clinical Findings, Imagery, Biology, Bacteriology, Treatment, and Outcome of 78 Children With Pyogenic Sacroiliitis, Based on a Recent Review of Literature

**Table 1 (Continued):**
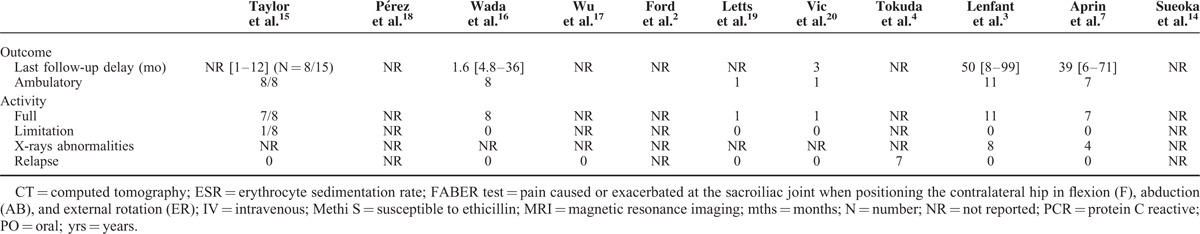
History, Clinical Findings, Imagery, Biology, Bacteriology, Treatment, and Outcome of 78 Children With Pyogenic Sacroiliitis, Based on a Recent Review of Literature

While hematogenous osteoarticular infections are common in children, pyogenic sacroiliitis is specially rare and mainly affects older children.^1–5^ It is quite exceptional at the age of 13 months. Only 3 cases of pyogenic sacroiliitis in children younger than 13 years have been reported in our review of the literature. They were aged of 9, 10, and 12 months.^3,4,14^

Onset to presentation of our patient was 4 days. It is likely similar to the delay we found in the literature since most patients consulted between 2 and 7 days after the beginning of the symptoms. The patient of the series of Lenfant et al^3^ who presented at 2 years had been unsuccessfully treated in another hospital before. It seems that the early age of our patient did not delay the onset to presentation.

In very young children, clinical and epidemiological characteristics complicate the diagnosis. Indeed, clinical examination is particularly difficult and sometimes does not help the clinician, particularly when the child does not speak yet. FABER test is rarely feasible. Moreover, children with pyogenic sacroiliitis consult most often for limping or decreased weight bearing. But these symptoms are very common reasons for consultation in young children and main etiologies are hematogenous osteoarticular infections localized to the knee or the hip and trauma. These difficulties may delay the diagnosis or refer misdiagnosis.^2,5,6,9,14^ Cases of patients who have been wrongly operated on for an appendicitis whereas they had a sacroiliitis have been reported.^21,22^

Bone scintigraphy is useful in young children to locate a bone or joint disease responsible for limping. In this observation, the combination of decreased weight bearing and inflammatory syndrome suspected an osteoarticular infection. It is the bone scintigraphy that located the infection at the right sacroiliac joint. The review of the literature confirms the high sensitivity of bone scintigraphy for the diagnosis of pyogenic sacroiliitis since it made the diagnosis in all but 2 of the 50 patients who had a bone scintigraphy.^2–4,7,14,17,19,20^ But given the unusually young age of our patient, we performed an MRI to confirm the diagnosis, to see the extent of inflammation, to search an etiology and to eliminate a complication such as an abscess. The review of the literature confirms that MRI has a good sensitivity for the diagnostic of pyogenic sacroiliitis since it was positive in all but 1 of the 16 patients who had an MRI.

The Staphylococcus aureus isolated in the joint aspiration is the main pathogen in our review of the literature since it represented 30 of the 41 pathogens identified. Prevalence of resistance to methicillin was not clearly detailed but it seemed that Staphylococcus aureus susceptible to methicillin was very more frequent.^15^ Other cases of sacroiliitis due to Staphylococcus epidermidis, Streptococcus (Groups A and pneumoniae), Citrobacter freundii, Salmonella spp., Bacillus subtilis, and *Escherichia coli* have been reported in children.^1,3,4,7,14,17,20,23,18^

Despite the very young age of the patient and the delayed start of treatment, symptoms rapidly disappeared with appropriate antibiotic therapy.

## CONCLUSIONS

This observation reports a very rare case of pyogenic sacroiliitis in a 13-month-old child. It reminds the risk of misdiagnosing pyogenic sacroiliitis in very young children because it is exceptional. It also highlights the difficulty to diagnose it in children and the usefulness of bone scintigraphy and MRI in osteoarticular infections in children.
