# Pyogenic Sacroiliitis in a 13-Month-Old Child: A Case Report and Literature Review: Erratum

**DOI:** 10.1097/01.md.0000476362.59481.90

**Published:** 2015-12-31

In the article “Pyogenic Sacroiliitis in a 13-Month-Old Child: A Case Report and Literature Review”,^1^ which appeared in Volume 94, Issue 42 of *Medicine*, the author's names were incorrectly transposed in the byline. The article has since been corrected online.
